# Comparison and development of cross-study normalization methods for inter-species transcriptional analysis

**DOI:** 10.1371/journal.pone.0307997

**Published:** 2024-09-10

**Authors:** Sofya Feldman, Hadas Ner-Gaon, Eran Treister, Tal Shay

**Affiliations:** 1 Dept of Computer Science, Ben-Gurion University of the Negev, Beer-Sheva, Israel; 2 Dept of Life Sciences, Ben-Gurion University of the Negev, Beer-Sheva, Israel; The First Hospital of Jilin University, CHINA

## Abstract

Performing joint analysis of gene expression datasets from different experiments can present challenges brought on by multiple factors—differences in equipment, protocols, climate etc. “Cross-study normalization” is a general term for transformations aimed at eliminating such effects, thus making datasets more comparable. However, joint analysis of datasets from different species is rarely done, and there are no dedicated normalization methods for such inter-species analysis. In order to test the usefulness of cross-studies normalization methods for inter-species analysis, we first applied three cross-study normalization methods, EB, DWD and XPN, to RNA sequencing datasets from different species. We then developed a new approach to evaluate the performance of cross-study normalization in eliminating experimental effects, while also maintaining the biologically significant differences between species and conditions. Our results indicate that all normalization methods performed relatively well in the cross-species setting. We found XPN to be better at reducing experimental differences, and found EB to be better at preserving biological differences. Still, according to our in-silico experiments, in all methods it is not possible to enforce the preservation of the biological differences in the normalization process. In addition to the study above, in this work we propose a new dedicated cross-studies and cross-species normalization method. Our aim is to address the shortcoming mentioned above: in the normalization process, we wish to reduce the experimental differences while preserving the biological differences. We term our method as CSN, and base it on the performance evaluation criteria mentioned above. Repeating the same experiments, the CSN method obtained a better and more balanced conservation of biological differences within the datasets compared to existing methods. To summarize, we demonstrate the usefulness of cross-study normalization methods in the inter-species settings, and suggest a dedicated cross-study cross-species normalization method that will hopefully open the way to the development of improved normalization methods for the inter-species settings.

## 1 Introduction

Gene expression profiling is a powerful tool for understanding biological phenomena at the molecular level. In recent years there has been significant increase in the volume of gene expression studies and publicly available gene expression datasets. A cross-study analysis of two (or more) gene expression datasets that profile the same conditions can increase the confidence in the identified differences and similarities between the conditions. Combining several datasets can also be used to train machine learning models, which benefit from using large amounts of data to get better results [1]. Additionally, an analysis of merged datasets may lead to a lower false discovery rate compared to a meta-analysis of the same datasets [2].

However, a combined analysis of data from several datasets raises difficulties, as even profiles of the same cell type under the same conditions can vary greatly in different datasets. These differences can occur due to the use of different platforms, protocols, etc. [3]. Naturally, an even greater challenge is posed when the analyzed datasets are from different species. Specifically, comparisons of human and mouse are of special interest, as a better understanding of the expression level differences between mice and humans may improve the translation rate from experiments in mice to experiments in humans, and reduce the number of experiments in both humans and mice that are based on irrelevant evidence. For example, if a tested drug targets a specific human protein, then performing the experiment on mice might offer no additional useful information if the gene encoding for this protein displays a different expression profile in mice compared to humans.

Attempting to make datasets more comparable by applying some transformation is called “cross-study normalization”, “harmonization” or “cross-platform normalization”. Such transformation translates two (or more) datasets to a comparable state by changing the values of the datasets to a similar scale and distribution, while significant differences among the values are conserved. The underlying assumption is that the real gene expression distribution is similar across conditions and datasets. The goal of the cross-study normalization is thus to reduce differences between the datasets that are caused by non biological (technical) factors. Existing cross-study normalization methods are mostly used on datasets from the same species, with a few exceptions [4].

A comparison of multiple cross-study normalization methods on two publicly available cross-platform testing datasets was performed in [5]. The Cross-Platform Normalization (XPN) method [6] displayed the best performance when treatment groups are of equal size. The Distance Weighted Discrimination (DWD) method [7, 8] was the most robust when the treatment groups are of different sizes. The Empirical Bayes (EB) method [9] which is very popular for within-study batch correction performed well on both scenarios.

**Fig 1:**
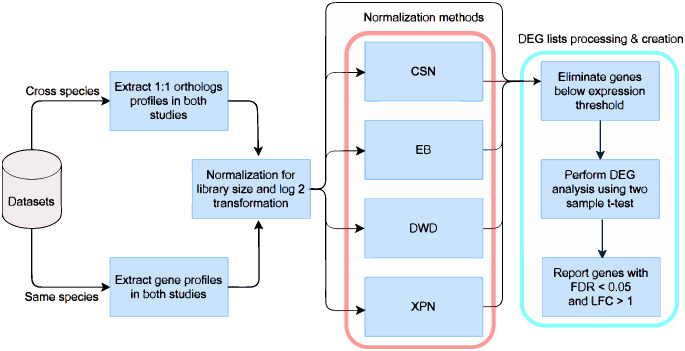
Flow chart of the analysis. DEG—differentially expressed genes; LFC—log fold change, FDR—false discovery rate.

Here we applied the three leading methods according to the above comparison for cross-study normalization—XPN, DWD and EB—to cross-species cross-study comparison. To evaluate the normalization methods we introduce cross-species performance evaluation criteria which test whether the normalization method only corrects technical differences, or also eliminates biological differences of interest. The pipeline of our approach is demonstrated in Fig 1. We show that the application of cross-study normalization methods on bulk RNA sequencing (RNA-seq) datasets from different species improves the comparability of the datasets. In addition, we develop a new cross-study cross-species gene expression normalization method (CSN), which has several advantages over the existing cross-study normalization methods in the cross-species context. In particular, we demonstrate that CSN leads to more predefined and balanced preservation of biological information in the normalized datasets.

## 2 Methods and data

### 2.1 Datasets

Four RNA-seq datasets of immune cells were used, two from mouse and two from human. For the purpose of this study, only naive immune cells were used, as it is harder to estimate if a stimulation is the same in two different experiments. The first mouse dataset is GSE122597 [10], which contains 83 samples of 5 naïve immune cell types.

The second mouse dataset is GSE124829 [11], from which only the 65 naive samples that passed quality assurance were used. This dataset contains 10 cell types: B, CD4 T cells (CD4), CD8 T cells, dendritic cells (DC), MF, natural killer T cells (NKT), natural killer cells (NK), neutrophils, regulatory T cells (Treg), and gamma delta T cells (Tgd).

The first human dataset is GSE60424 [12], of which we used 20 healthy control samples of six cell types, monocytes (MO), B, CD4 T cells, CD8 T cells, and NK. The second human dataset is GSE107011 [13], which contains 127 samples of which 28 samples of naive cells from 8 cell types were used in this study: B, MO, CD8 T cells, CD4 T cells, DC, NK, and Treg.

#### 2.1.1 One-to-one orthologous genes

A list of orthologous genes between mouse and human was downloaded from Ensembl Genes version 104 with BioMart data mining tool [14] from the following link: http://www.ensembl.org/biomart/martview. Only one-to-one orthologous genes were used.

### 2.2 Data pre-processing

All datasets were downloaded from NCBI GEO. Mouse datasets were already deposited with read count tables, which were used as is. For the human datasets, no read count tables were deposited. Thus, RNA sequencing reads were mapped to the human genome (hg38) using hisat2 [15] (version 2.0.5). BAM files were sorted and indexed by SAMtools [16] (version 1.9). Expression counts at the gene level were obtained by FeatureCount [17] (version 2.0.1). All raw read counts datasets were normalized for library size and zero values were replaced by 1. Then, all the datasets were log2 transformed, as all normalization methods used expect log2(read counts) as input. Normalization methods on datasets from two different species were applied only to one-to-one orthologous genes between the two species.

### 2.3 Application of the normalization methods

We applied XPN, DWD and EB normalization methods on the datasets described above. All genes which do not appear in both datasets of the same species were omitted before applying any normalization method. When normalization was applied on datasets from different species, it was applied only on one-to-one orthologous genes.

The code for the XPN method was taken from https://genome.unc.edu/xpn. DWD was run through the CONOR package [5] which is available in: https://github.com/jcrudy/CONOR. The code used for the EB method was applied using the ComBat function from the SVA package (https://bioconductor.org/packages/release/bioc/vignettes/sva/inst/doc/sva.pdf). The input for this function is an expression matrix and a batch vector. We merged every two datasets to one expression matrix and generated a proper batch vector that indicates which sample belongs to which dataset. The expression matrix must not include any zero rows, thus, we removed the genes that are not expressed in any of the samples before applying the EB method and attached those genes with the same zero values to the output. We applied the code of these methods with default parameters on every two datasets.

Normalization for same species datasets was applied between mice datasets GSE122597 and GSE124829, where the number of the common genes was 49468, and between human datasets GSE60424 and GSE107011, where the number of the common genes was 45131. Cross-species normalization was applied between all pairs of human-mouse datasets mentioned above. The mouse datasets had 15877 common 1:1 orthologous genes with human dataset GSE60424, and 16444 1:1 orthologous genes with the human dataset GSE107011.

### 2.4 Identifying differentially expressed genes

To obtain lists of differentially expressed genes (DEGs), we used the mattest MATLAB function with default values (https://www.mathworks.com/help/bioinfo/ref/mattest.html), which performs two-sample t-test assuming the two samples have unknown and unequal variances. The mafdr MATLAB function, which estimates the false discovery rate (FDR), was applied to the output p-values achieved by the t-test. The log fold change (LFC) was calculated for each gene for any two groups. Before applying these calculations on any two groups, for example, *H*_*C*1 and *M*_*C*2 described below, all genes with expression levels that did not pass the noise threshold in at least *n* samples (where *n* represents the size of the smallest group) were filtered out. The noise threshold for all datasets was set to 4 (after log2 transformation). After applying the calculations, genes with FDR>0.05 or an absolute value of the LFC lower than 1 were filtered out, to increase the reliability of the DEGs.

### 2.5 The performance evaluation method

**Fig 2:**
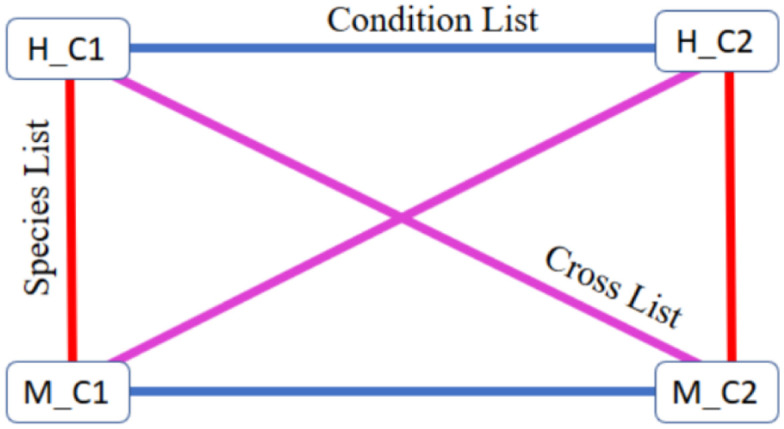
Scheme of differentially expressed gene lists in a cross-species normalization setting. Every square represents a sample group: *H*_*C*1—samples of condition *C*1 from experiment *H*, *M*_*C*1—samples of condition *C*1 from experiment *M*, *H*_*C*2—samples of condition *C*2 from experiment *H*, *M*_*C*2—samples of condition *C*2 from experiment *M*. Every line represents a list of differentially expressed genes between these groups. Condition lists (blue) represent the biological difference between C1 and C2 in each species. Species lists (red) represent the difference between the same condition in different species, and cross lists (purple) represent the difference between different conditions from different species. Ideally, each cross list is the union of the species and the condition lists.

In order to determine how well a normalization method corrects the cross-study effects, the following evaluation process is carried out:

Let *H* be an experiment on one species (here, humans) and *M* an experiment on another species (here, mice). After applying normalization on these datasets, we select two conditions that are profiled in both experiments, termed C1 and C2, and produce lists of DEGs. Any method for identifying DEGs can be used. Here we used two-sample t-test and filtered by FDR correction and LFC values. The following lists of DEGs are then being generated (illustrated in Fig 2)

1. *H*_*C*1 − *H*_*C*2: DEGs in experiment *H* between C1 and C2—condition list.
2. *M*_*C*1 − *M*_*C*2: DEGs in experiment *M* between C1 and C2—condition list.
3. *H*_*C*1 − *M*_*C*2: DEGs between C1 in experiment *H* and C2 in experiment *M*—cross list.
4. *H*_*C*2 − *M*_*C*1: DEGs between C1 in *M* and C2 in *H*—cross list.
5. *H*_*C*1 − *M*_*C*1: DEGs between C1 in *H* and C1 in *M*—species list.
6. *H*_*C*2 − *M*_*C*2: DEGs between C2 in *H* and C2 in *M*—species list.

Given these lists, we say that a normalization method is better for cross-species normalization if it performs well according to the following two criteria.

#### 2.5.1 Criterion 1: The condition lists and the cross lists should be similar while the cross lists should differ from the species lists

In other words, this criterion indicates that there should be large intersections between cross and condition lists which will indicate similarity. Large intersections between cross and species lists might indicate differences that occur mostly because of technical noise. Thus, optimal normalization should result in minimal intersections between cross and species lists. We would like to evaluate this in a quantitative way. To this end, we propose the following measurement: Let *P*_*ij*/*i*_ be the proportion of genes from list *i* that appear in the intersection with list *j* where *i* ≠ *j*:

$$\begin{array}{c} P_{ij/i}=\frac{|List_{i}\cap List_{j}|}{|List_{i}|}. \end{array}$$ (1)

Note that for each intersection there are two such values, *P*_*ij*/*i*_ for list *i* and *P*_*ij*/*j*_ for list *j*. Hence, there are 12 *P*_*ij*/*i*_’s—two for each of the six pairwise intersections of cross and condition lists as shown in Fig 2. Given the proportions defined in Eq (1), we define the Cross-Condition Average (CCA) index as the average of the *P*_*ij*/*i*_’s of all cross and condition lists pairwise intersections:

$$\begin{array}{c} CCA=\frac{\sum_{i,j=1\ldots 4,i\neq j}P_{ij/i}}{12}. \end{array}$$ (2)

Furthermore, we define the Species Average (SA) index as the average of the *P*_*ij*/*i*_ values of every species list with the five other lists, i.e. nine intersections and two *P*_*ij*/*i*_ for each intersection:

$$\begin{array}{c} SA=\frac{\sum_{i=1\ldots 4,j=5,6}\left(P_{ij/i}+P_{ij/j}\right)}{16}. \end{array}$$ (3)

An optimal normalization method will remove the technical differences, thus maximizing the overlap between cross and condition lists. Ideally, the cross list should include most of the genes in the condition list, with a few genes that are differentially expressed between species added or removed. The CCA index expresses the level of that overlap. Large overlap will result in high CCA index. On the other hand we expect that for an optimal normalization method, each species list will have a small overlap with condition and cross lists, i.e. low SA index.

We combine both indices in (2) and (3) to one, defining the Cross-Study Comparison (CSC) index

$$\begin{array}{c} CSC_{\text{normalization}}\left(X,Y\right)=\frac{CCA(X,Y)}{SA(X,Y)}, \end{array}$$ (4)

where *X*, *Y* are the two datasets after the normalization. This index is a combination of the previous two indices and it reflects how good a normalization has performed according to the two criteria above. A high CSC index indicates that the normalization has preformed well.

The rationale of the first criterion is that the biological differences between the two conditions were already in place in the last common ancestor of the two species. Thus we assume that the DEGs between the two conditions within each species mostly overlap between species. On the other hand, without the reduction of technical noise, the cross lists will have more genes and will have a large overlap with the species lists because they will include mostly the genes that have differences caused by technical reasons.

#### 2.5.2 Criterion 2: The condition lists should not be affected by normalization

In other words, this criterion indicates that *H*_*C*1 − *H*_*C*2 and *M*_*C*1 − *M*_*C*2 that were obtained between conditions in the same dataset will be similar with and without applying any normalization. To evaluate how good a normalization method performs according to this criterion, we use the Intersection Over Union (IOU) index, which is the ratio between the intersection size and the union size of the condition lists before and after applying normalization method:

$$\begin{array}{c} IOU_{\text{normalization}}\left(dataset\right)=\frac{|cond\;list\cap cond\;list_{\text{after}\;\text{normalization}}|}{|cond\;list\cup cond\;list_{\text{after}\;\text{normalization}}|}. \end{array}$$ (5)

The ideal situation is where the DEGs between the two conditions within a dataset will stay the same as before any normalization method was applied, resulting in *IOU* = 1. Optimal normalization should neither add DEGs nor erase differences between the conditions. IOU index range is [0 1], where values that are closer to 1 reflect that the normalization performed well according to the second criterion.

The second criterion makes certain the original difference between cell types resembles the difference between cell types after normalization, thus confirming biological differences between conditions remain unchanged after applying normalization.

### 2.6 Optimization-based normalization using a CSC-IOU loss

All cross-study normalization methods known to us were designated for datasets of the same species. Thus, those methods include an assumption that the gene expression is similar across studies. In our case, different species might have different expression levels of orthologous genes, which may be captured as cross-study effect during the normalization. Thus, existing methods might adjust the expression levels of these genes between dataset to be similar. This may cause unwanted alteration to the datasets’ information. For example, differentially expressed genes between two subgroups of the same dataset before normalization may have similar expression level after normalization. To reduce this effect, we would like to improve the IOU index defined in Eq (5), which determines how much the data is distorted by the applied normalization.

Now we define our proposed approach at cross-study and cross-species normalization (CSN) method, which aims to directly improve our IOU and CSC indices. To this end, we first choose a parameterized displacement model, i.e., a technique that adjusts the datasets to comparable scales given some parameters that we compute. That is similar to existing methods, like XPN and EB for example. Then, we define an objective function (a loss) to be minimized based on a combination of the IOU and CSC indices of the displaced datasets. The objective is minimized with respect to the displacement parameters. Since the loss is defined based on the IOU and CSC indices, reducing it guarantees that we preserve biological information on one hand, and on the other hand we make the datasets more comparable. In particular, our approach has the ability to set a constraint on the IOU, i.e., we can control the amount of lost data by defining the lowest allowed IOU rate for the datasets after normalization. This feature is important as current methods do not have this direct ability, and one has to hope or carefully fine-tune each of the other method’s parameters in an attempt to control the IOU.

The description below is a general recipe that we propose to tackle the cross-platform cross-species normalization:

1. Choose a parameterized displacement model.
2. Choose a loss (or, a combination between IOU and CSC).
3. Use a derivative-free optimization to find the optimal parameters.

We demonstrate the approach with specific choices below, but other components may be equally suitable for our framework.

#### 2.6.1 The displacement model

Our proposed displacement method adjusts the two datasets towards each other. That is, given two datasets *X* and *Y*, the displacement model adjusts both dataset *X* and dataset *Y*. In a nutshell, we first cluster the genes and perform a simple standardization. Then we find the intercept and slope parameters for each of the gene clusters using an optimization method. We cluster the genes in order to reduce the number of parameters in the displacement model, where each cluster of genes is displaced by two parameters. In order to cluster the genes we merge *X* and *Y* to one dataset and cluster the merged gene vectors using fuzzy k-means algorithm [18]. In our implementation, we have *k* = 50 gene clusters.

After the clustering is obtained, we apply our displacement method, which contains two steps: standardization and displacement by clusters. The standardization is performed by first computing new means and standard deviations for both datasets:

$$\begin{array}{ccc} \mu_{x}^{st} & = & \frac{w_{x}^{x}\cdot \mu_{x}+w_{y}^{x}\cdot \mu_{y}}{w_{x}^{x}+w_{y}^{x}},\;\sigma_{x}^{st}=\frac{w_{x}^{x}\cdot \sigma_{x}+w_{y}^{x}\cdot \sigma_{y}}{w_{x}^{x}+w_{y}^{x}}\\ \mu_{y}^{st} & = & \frac{w_{y}^{y}\cdot \mu_{y}+w_{x}^{y}\cdot \mu_{x}}{w_{y}^{y}+w_{x}^{y}},\;\sigma_{y}^{st}=\frac{w_{y}^{y}\cdot \sigma_{y}+w_{x}^{y}\cdot \sigma_{x}}{w_{y}^{y}+w_{x}^{y}}, \end{array}$$ (6)

where *μ*_*i*_ is a gene-wise mean vector and *σ*_*i*_ is a gene-wise standard deviation vector. The parameter $w_{i}^{j}$ is the standardization weight multiplier of dataset *i* for the transformation of dataset *j* that our algorithm aims to find. Given the new means and standard deviations, the datasets are standardized as follows:

$$\begin{array}{c} X_{st}=\frac{X-\mu_{x}}{\sigma_{x}}\cdot \sigma_{x}^{st}+\mu_{x}^{st},\;Y_{st}=\frac{Y-\mu_{y}}{\sigma_{y}}\cdot \sigma_{y}^{st}+\mu_{y}^{st} \end{array}$$ (7)

The standardization occurs element-wise. From each entry (*X*)_*gs*_ or (*Y*)_*gs*_, where *g* is the gene and *s* is the sample, the mean value for this gene *g* in the dataset is subtracted and then divided by the standard deviation for this gene *g*.

Next, the cluster displacement step is done for the *k* clusters on the standardized data. We would like to find the best intercept and slope parameters for each cluster for every dataset on *X*_*st*_ and *Y*_*st*_, i.e. for each entry clustered in cluster *i*, its new value in *X*_*new*_ is:

$$\begin{array}{c} {\left(X_{new}\right)}_{gs}={\left(X_{st}\right)}_{gs}\cdot \alpha_{i}^{x}+\beta_{i}^{x}\\ {\left(Y_{new}\right)}_{gs}={\left(Y_{st}\right)}_{gs}\cdot \alpha_{i}^{y}+\beta_{i}^{y} \end{array}$$ (8)

where $\alpha_{i}^{j}$ is the slope parameter found for cluster *i* in dataset *j* and $\beta_{i}^{j}$ is the intercept parameter found for cluster *i* in dataset *j*.

#### 2.6.2 The loss function

The evaluation of how well a transformation is performed can be utilized by the performance evaluation method, as described below. We want to extract the significant values of our performance evaluation method described in Section 2.5, and merge them into one value that will serve as our loss value. We chose to use the following values:

- CSC—we used the CSC index calculated for the two datasets. In principle, we wish to maximize the CSC value, hence we take this index with a minus sign for our loss.
- IOU—we extracted the IOU of each dataset. This helps us with the understanding of how bad a normalization distorted the biological information within each dataset by erasing the original DEGs, or by adding non-existing DEGs in the condition list.

Thus, our evaluation loss is comprised of three elements for each normalization:

$$\begin{array}{ccc} F(X,Y) & = & -CSC(X,Y)-IOU(X)-IOU(Y)+\\ & & m\cdot (max{\left(t-IOU\left(X\right),0\right)}^{2}+max{\left(t-IOU\left(Y\right),0\right)}^{2}) \end{array}$$ (9)

where *X* and *Y* are the translated datasets, *t* is the minimal threshold for the desired IOU value for both datasets, and *m* is the multiplicative penalty parameter, which weighs the penalty term that limits the decrease of the IOU so that it is not far below the threshold parameter at the lowest. For *t* = 0 it means that penalty is meaningless, i.e. 0, and there is no constraint on the minimal value of the IOU for the normalized datasets. Setting *t* > 0 means that we normalize the dataset with minimal IOU of *t*.

Given *k* clusters of genes, which in our case were established using fuzzy c-means algorithm, the loss function we defined gets 4*k* + 4 parameters—2*k* + 2 parameters for each dataset. For every dataset, these 2*k*+ 2 parameters include two weight parameters for the standardization in Eq (6), and the intercept and slope for each cluster in Eqs (7) and (8). The function returns the F evaluation value for the dataset after the displacement technique was applied using these 2 ⋅ (2*k* + 2) = 4*k* + 4 parameters.

#### 2.6.3 Gradient-free optimization

After defining the loss function, we would like to apply an optimization algorithm to minimize the loss function, and find the best parameters for our displacement method. Our objective function F is based on DEG analysis done by two-sample t-test with FDR correction and calculating the LFC values. Because this function is not easily differentiable, we treat it as a black-box, and use a gradient free optimization algorithm to minimize the loss. The algorithm that we chose for this purpose was CMA-ES [19].

The MATLAB code of the CMA-ES algorithm was taken from [20]. This code requires a range for the parameters to be found (these are *α*_*i*_ and *β*_*i*_ in Eq (8)), and a maximum number of iterations. In our case, the search range was [−2, 2] around the centers *α*_*i*_ = 1 and *β*_*i*_ = 0. The maximum number of iterations was 50, and since we use *k* = 50 clusters, the number of parameters was 4*k* + 4 = 4 * 50 + 4 = 204. The number of clusters was set to 50 after trial and error. It should be mentioned that for other number of clusters, e.g., in the range 20-80, the results did not changed dramatically. We also made some changes to the default parameters in the code:

1. During the initialization, the step size *σ*^(0)^ was set to *σ*^(0)^ = 0.01 ⋅ (*VarMax* − *VarMin*) instead of *σ*^(0)^ = 0.3 ⋅ (*VarMax* − *VarMin*) where *VarMax* = 2, *VarMin* = −2.
2. The solution was initialized to be a zero change for all the parameters. That is, *α*_*i*_ = 1, *β*_*i*_ = 0 for all clusters, $w_{x}^{x}=w_{y}^{y}=1$, and $w_{y}^{x}=w_{x}^{y}=0$.

The first change (numbered 1.) was made to set the step size to the scale of our problem.

Finally, we applied the CMA-ES algorithm with our objective function. Once the CMA-ES finished, the 4*k* + 4 parameters that were found were applied according to the standardization and displacement method in Eqs (7) and (8).

## 3 Results

In this section we present results that demonstrate our performance evaluation method for assessing the performance of the existing normalization methods, and for our CSN method with different parameters as detailed later. In this section we compare our method only to EB and XPN, as they met the evaluation performance criteria to a greater extent compared to DWD, and another naive normalization method that we developed. The results for these two other methods are qualitatively similar and are shown in Appendix A.

All the normalization methods were first applied to two datasets of the same species—human or mouse. Then, we applied cross-species normalizations on a human dataset and a mouse dataset. The cell types that were chosen to demonstrate the effect of cross-species normalization are B cells and CD4 T cells, referred to as B and CD4, which were profiled in all four datasets.

**Fig 3:**
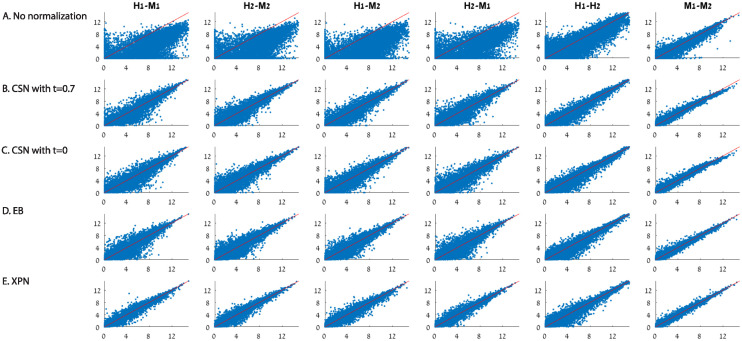
Scatter plot similarity comparison before and after normalization. Comparison of overall similarity of samples log2 expression values before and after applying normalization methods using mean-mean scatter plots. Mean-mean scatter plots of naïve CD4 T cells from different datasets: H1 is the human dataset GSE60424, M1 is the mouse dataset GSE122597, H2 is the human dataset GSE107011 and M2 is mouse dataset GSE12482. (A) No normalization (B) CSN with *t* = 0.7 (C) CSN with *t* = 0 (D) EB (E) XPN. In each scatter plot the mean vector of the first dataset is on the X axis and the mean vector of the second dataset is on the Y axis. The red line is the *y* = *x* equation. Axes represent log2 expression values.

Fig 3 shows representative scatter plots of several samples before and after each normalization. The scatter plots demonstrate that all the considered normalization methods increased the similarity between the data sets, as reflected in the reduction of the distribution width of the genes around the main diagonal. In Fig 3B and 3C we present the datasets after CSN: every combination of two datasets were normalized twice—once by setting the minimal IOU threshold parameter *t* in Eq (9) to 0.7, and once without any constraint on the minimal value of the IOU, i.e. *t* = 0. The scatter plots in Fig 3B and 3C display an increase in the similarity between the samples compared to the scatter plots from the datasets before normalization in Fig 3A. Qualitatively we see that both of the CSN normalizations, with or without a constraint on the minimal value of IOU, performed quite well.

Next, we compared the DEGs lists before and after applying normalization methods on datasets from different species and on datasets from the same species.

**Fig 4:**
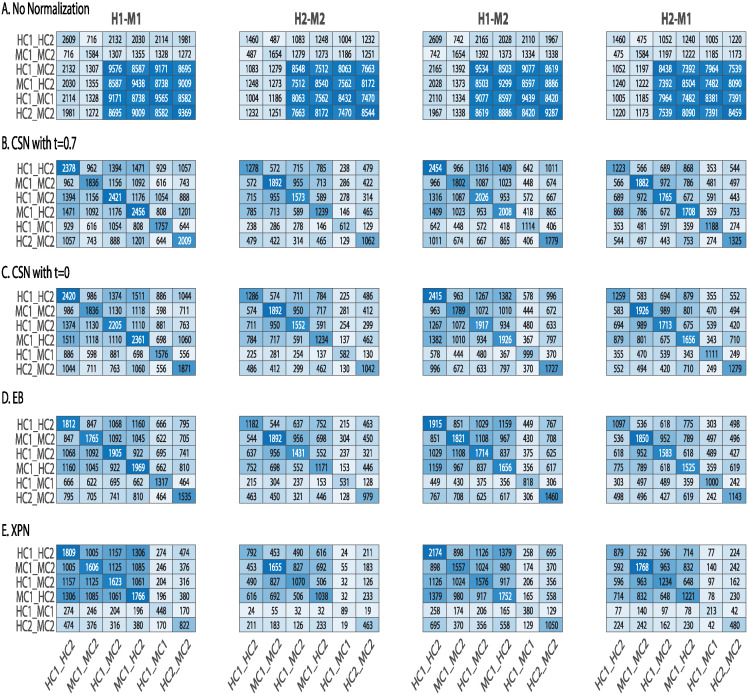
Comparison of DEGs lists before and after application of normalization methods. Heatmaps presenting the size of the intersection between lists of differentially expressed genes before (A) and after applying the following normalization methods on cross-species datasets: (B) CSN with *t* = 0.7 (C) CSN with *t* = 0 (D) EB (E) XPN. Shown is the intersection size between the species lists, the condition lists and the cross lists. The diagonal entries are the intersection size of each list with itself, i.e. every diagonal entry (i,i) is the number of genes in list i. Entry (i,j) represents the number of genes in the intersection of list i with list j. H1 is the human dataset GSE60424, M1 is the mouse dataset GSE122597, H2 is the human dataset GSE107011 and M2 is the mouse dataset GSE124829. The condition C1 is CD4 T cells and the condition C2 is B cells.

In Fig 4 we show the sizes of the lists and the intersections between them before normalization (Fig 4A) and after normalization (Fig 4B–4E). We see that after normalization on datasets from different species, the sizes of the cross lists and species lists are much smaller, and the size of condition lists is similar for CSN (Fig 4B and 4C) or somewhat smaller for EB and XPN (Fig 4D and 4E) than without normalization.

**Fig 5:**
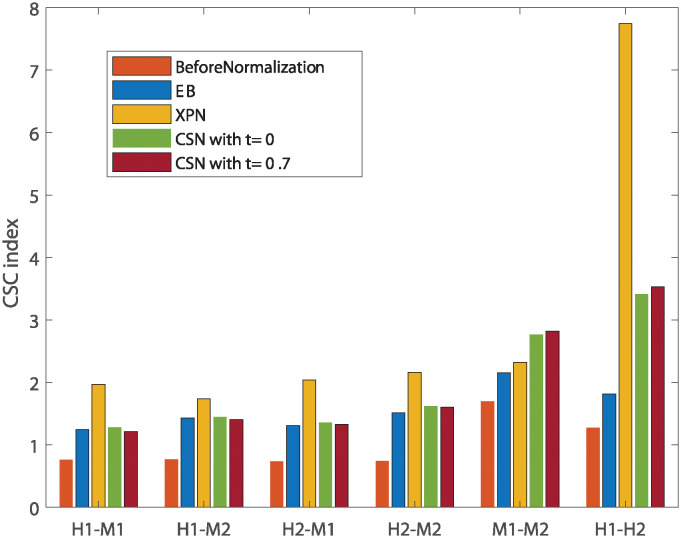
Comparison of performance of normalization methods according to the CSC index. Every bar represents the CSC index for a specific comparison under a specific normalization method applied on the two datasets. H1 is the human dataset GSE60424, M1 is the mouse dataset GSE122597, H2 is the human dataset GSE107011 and M2 is the mouse dataset GSE124829.

Fig 4B and 4C show smaller size of the cross and species lists after application of the CSN method. It can be seen that the size of the condition lists in these tables is quite conserved. It is also noticeable that there is an increase in the intersection size of cross lists and condition lists, which is displayed in the upper left relatively dark blue square in Fig 4B and 4C. The upper left square looks darker in Fig 4C than in Fig 4B. This is also reflected in higher CSC index for normalization with *t* = 0 than with *t* = 0.7 as presented in Fig 5 that compares the CSC values for all methods. We assume this is happening because the CSC and the IOU are negatively correlated, i.e., the higher the IOU, the lower the CSC and vice versa. This relation indicates that there is a trade-off between conserving the signal making the datasets more similar. Fig 5 also shows that the performance of the CSN method was similar and sometimes even better in terms of similarity between the datasets according to the CSC index.

**Fig 6:**
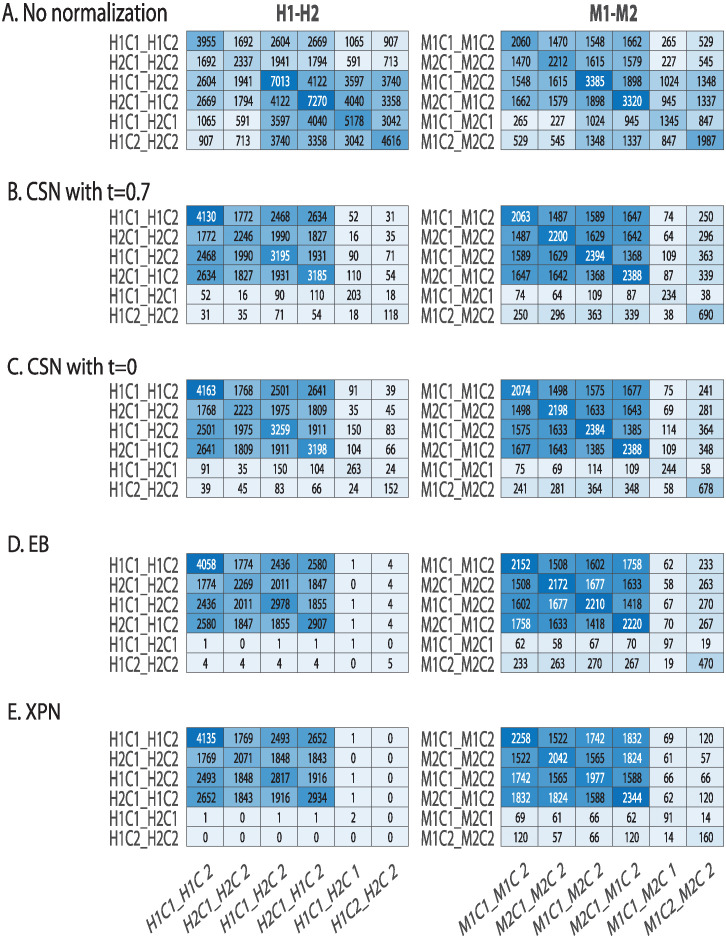
Comparison of lists of differentially expressed genes before and after applying normalization methods on same species datasets. (A) No normalization, (B) CSN with *t* = 0.7 (C) CSN with *t* = 0 (D) EB (E) XPN. Shown is the intersection size between the species lists, the condition lists and the cross lists. The diagonal entries are the intersection size of each list with itself, i.e. every diagonal entry (i,i) is the number of genes in list i. Entry (i,j) represents the number of genes in the intersection of list i with list j. H1 is the human dataset GSE60424, M1 is the mouse dataset GSE122597, H2 is the human dataset GSE107011 and M2 is mouse dataset GSE124829. The condition C1 is CD4 T cells and the condition C2 is B cells.

The application of all the methods (CSN, EB, and XPN) on same species datasets display an increase in similarity between the datasets demonstrated by a dark square on the top left of Fig 6B–6E. These results are also shown in Fig 5 by using the CSC index defined in Eq (4).

It seems that XPN displays the largest intersection size between the condition and cross lists contrary to smaller intersections of species lists with the rest of the lists. On the other hand, EB and caused a larger intersection size of species lists with the rest of the lists, meaning these shared genes could be indicative of noise that was not removed. Thus, we estimate that XPN has satisfied the first criterion to a greater extent.

On datasets from the same species the leading methods and the CSN behaved as expected, i.e. high CSC index and a relatively dark blue square on the top left in Fig 6B–6E.

Fig 6B and 6C look quite the same. This is reasonable because the constraint on the minimal IOU value did not affect datasets of the same species because the IOU of these datasets were over 0.7 in the first place. This means that the normalization behaved almost identically with or without threshold on the minimal IOU.

**Fig 7:**
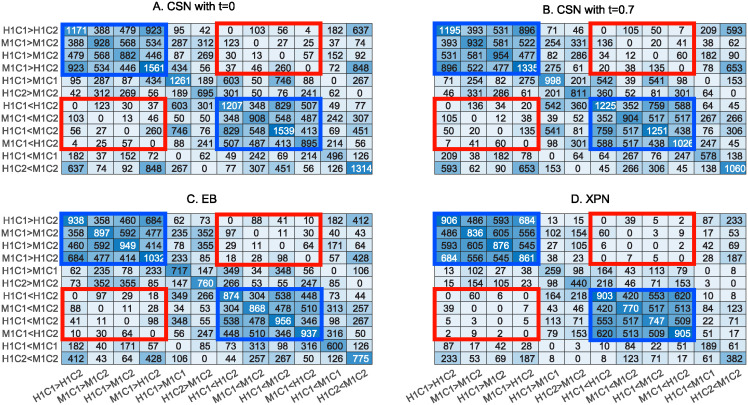
Comparison of lists of DEGs after applying four normalization methods, split by the direction of change. (A) CSN with t = 0.7, (B) CSN with t = 0, (C) EB and (D) XPN. Note that each list (row and column) that appeared in Fig 4 is split into two lists here (two rows and two columns). Blue squares mark the rectangles of overlap between condition or cross lists in the same direction. Red squares mark the rectangles of overlap between condition or cross lists in the opposite direction.

We hypothesize that genes which randomly or due to experimental factors came out as DEGs, are as likely to change direction in each comparison as they are to differ in the same direction, whereas genes that are biologically different between conditions will probably change in the same direction in all datasets. To evaluate what fraction of the DEGs corresponds to noise and technical factors and what fraction corresponds to biological signal, we split the lists of DEGs by direction of change in expression. For example, the list *A*_*i*_−*B*_*j*_ was separated into two lists, *A*_*i*_ > *B*_*j*_, where genes have higher expression in *A*_*i*_ than in *B*_*j*_, and *A*_*i*_ < *B*_*j*_ where genes have lower expression in *A*_*i*_ than in *B*_*j*_. Reassuringly, the two darker squares in Fig 7B–7D (marked blue) indicate that there is a big overlap of the condition and cross lists of the same direction, and almost no overlap between the condition and cross lists of the opposite direction (light squares, marked red). As expected, there is an overlap between cross lists and species lists, because the cross lists also include the species differences. But, here too, the direction is consistent. This indicates that XPN, EB and CSN meet the first criterion. Again, XPN seems to remove the experimental effect better, potentially at the cost of removing some of the differences between the species.

**Fig 8:**
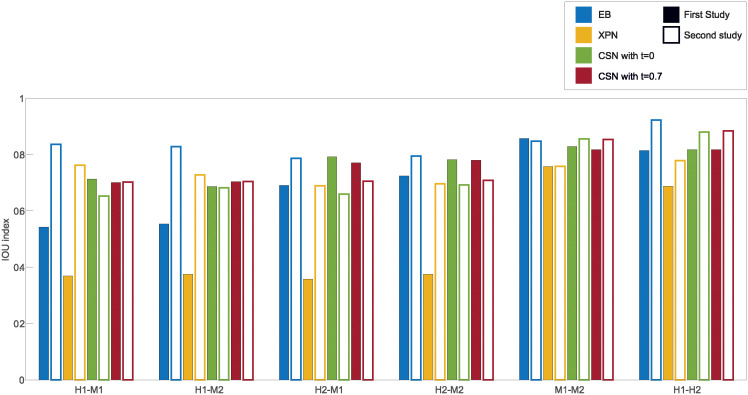
Comparisons of performance of normalization methods according to the IOU index. Every bar represents the IOU index for a condition list after normalization. A full bar represents the condition list from the first study and an empty bar represents the condition list from the second study. H1 is the human dataset GSE60424, M1 is the mouse dataset GSE122597, H2 is the human dataset GSE107011 and M2 is the mouse dataset GSE124829.

**Fig 9:**
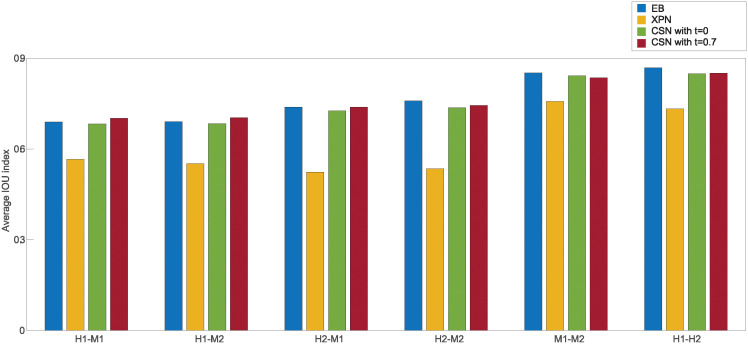
Comparison of performance of normalization methods according to the average IOU index. Every bar represents the average of the IOU index for the two condition lists after normalization. H1 is the human dataset GSE60424, M1 is the mouse dataset GSE122597, H2 is the human dataset GSE107011 and M2 is the mouse dataset GSE124829.

To test the second criterion, we compared every list of DEGs within dataset before and after normalization by calculating the IOU index. This comparison, which is shown in Fig 8, indicates whether a normalization caused loss of information by reducing the perceived biological difference between conditions within dataset. By doing that, we can estimate if a transformation done on a dataset to make it comparable to another dataset erased essential information. Fig 8 shows the IOU rate of the condition lists after each of the normalization methods was applied. The IOU index helps us understand the reduction of the biological difference between conditions within dataset, i.e. how much a normalization erased essential information. It is quite noticeable that in both XPN and EB there exists a large difference between the two IOU values of each dataset in some of the cross species normalization results. Contrary to this, our CSN method, both with or without setting minimal threshold on the IOU, achieves similar reduction of information in both datasets, and in fact never got an IOU value bellow 0.68 in any list. By looking at the average of the IOU values of every normalization shown if Fig 9, it is clear that our CSN method acts similarly to the EB method with *t* = 0 and in the cross species normalizations it is mostly better than the other methods with *t* = 0.7 in terms of the average reduction of information in both normalized datasets.

It appears that setting the minimal threshold of the IOU might have a large impact on the results. In addition to *t* = 0.7, we also normalized the cross species datasets with *t* = 0.75. The difference between *t* = 0.7 and *t* = 0.75 may look negligible, but has a large effect on the results. That is due to the high penalties incurred by the IOU index which measures not only the reduction of the number of DEGs in a condition list, but also the increase in the number of DEGs that did not appear in the original data. In Table 1 we show a comparison between the CSC and average IOU indices achieved by normalizing the datasets with *t* = 0.7 and *t* = 0.75. It is clear that small increase in the *t* parameter might have a large impact on the similarity achieved by the normalization. This emphasises the trade-off between CSC and the IOU, i.e. between the similarity and the distortion of the original data. We compared only cross species normalization due to the high IOU index in same species datasets—above 0.8. In Appendix B we show the objective history of the CMA-ES algorithm.

**Table 1:** CSC and average IOU values of the new normalization method.

CSC and IOU values for normalizations				
Indices for *t* values	H1-M1	H2-M2	H1-M2	H2-M1
CSC for CSN with *t* = 0.7	1.2105	1.6008	1.4043	1.3273
CSC for CSN with *t* = 0.75	1.012	1.5876	1.161	1.2321
Average IOU for CSN with *t* = 0.7	0.7016	0.74455	0.70385	0.73835
Average IOU for CSN with *t* = 0.75	0.7525	0.755	0.75205	0.76325

## 4 Discussion

Many comparative analyses in biology require comparison of datasets from different studies and different species. There are several methods for cross-study normalization of gene expression datasets. However, there is no method for cross-species normalization of gene expression datasets as far as we know. As cross-study normalization methods are designed with the assumption that differences between datasets are due to experimental factors, these methods aim to eliminate differences between datasets. However, whereas technical differences affect hundreds of genes in a similar fashion, biological differences between species are qualitatively different from experimental differences, and mostly affect a small number of genes. Thus, we tested if it is possible for such normalization methods to remove most if not all technical noise without eliminating biological differences between conditions and species.

We proposed a new optimization-based approach for cross-study and cross-species normalization. That is, using the ingredients of our performance evaluation method we defined an objective function that includes a displacement model and a loss value, and applied a gradient-free optimization algorithm to minimize it.

We applied our new normalization method, CSN, on human and mouse gene expression datasets, and then applied our performance evaluation method on these normalized datasets. The results show that our CSN method might cause a reduction in the technical noise according to the CSC index, which represents the similarity between the datasets, in both within species normalization and also between species. We also show the IOU rates, which represent the conservation of the biological differences within conditions in the dataset, for the normalized datasets. When we look at the loss of information after normalization between species, it seems that CSN performed better than the existing methods, mainly because of the similar loss in both of the datasets and low loss in general compared to other methods, with or without setting minimal IOU to the datasets.

The results also show the significance of choosing the *t* parameter which is responsible for the minimal IOU value of every dataset in the normalization. According to the results, without limiting the amount of the distortion of the datasets, i.e. *t* = 0, the normalization achieves higher CSC index than the EB method. When we applied CSN with *t* = 0.7, the normalizations that previously achieved IOU similar or higher than 0.7 behaved similarly, while the others, who were affected by the penalty, caused a reduction in the CSC index. This phenomenon was consistent when the value of *t* was set to 0.75. This indicates a trade-off between making datasets more similar and erasing biological information.

In our opinion, the ability to tune the minimal distraction of the datasets is a significant advantage. This enables to normalize the datasets in such manner that on one hand if making datasets more comparable has higher priority *t* can be set to lower value or even zero, and on the other hand, the minimal distraction of the original datasets can be set as high as desired.

Another advantage of the approach we propose is that given other metrics and other displacement methods, it will be possible to generate a new method based on them. That opens a door to new possible normalization methods that will improve the understanding of differences between species using existing gene expression datasets.

We also examined three often-used cross-study normalization methods, EB, XPN, DWD, and another simple baseline method (the results concerning the latter two methods are given in Appendix A). We note that not any cross-platform normalization method is applicable for cross-species. For example, the recent MatchMixeR [21] requires matched samples from different platforms, which is naturally impossible in cross-species settings.

As expected, applying normalization methods improves the capability to directly identify DEGs between species. After applying the leading normalization methods (XPN, DWD and EB) and CSN, there were more DEGs between conditions than between species, and most of the DEGs between conditions that are common to both species changed in the same direction. All methods identify more species DEGs for B cells than for CD4 T cells. This can be due to CD4 T cells being more conserved between human and mouse compared to B cells. Alternative explanations can be related to different sorting schemes of the two cell types between the two species, or homogeneity level of what is defined as the cell type in each species.

Applying the baseline method achieved a small reduction in the number of DEGs in the cross lists and the species lists. In addition, the size of the intersections between these lists were noticeably larger than the intersections between cross and condition lists, similar to intersections before normalization. This indicates that although the baseline method corrects for scale factor, it is not effective in eliminating technical noise.

Among the leading methods, our analysis show that XPN is best at reducing experimental noise, but may also reduce species differences while doing so. The EB performance was similar to the CSN method but better then XPN in terms of preserving biological differences, but on the other hand, caused a smaller reduction in cross-study effects then XPN and CSN. Notably, in both EB and XPN, the fraction of the overlap between the human and mouse DEGs of the two conditions is larger than the fraction of the overlap without normalization. This strengthens the advantage of applying normalization methods, over the often used heuristics of just comparing DEGs lists.

To conclude, application of cross-study normalization methods can be useful for comparison of datasets from different species, as long as the profiled conditions are similar. In the future, we will extend our performance evaluation of cross-species cross-study normalization method to all genes, and to multiple species. After evaluating the required correction between species using the 1:1 orthologs, the calculated correction will be applied to the entire datasets. In order to apply the cross-species normalization and comparison to more than two species, we will incorporate consideration of evolutionary distance.

## 5. Appendix

### A Studies using additional normalization methods

In addition to XPN and EB, as part of this study, we also examined the third leading method of this field which is distance-weighted discrimination (DWD) [7]. Furthermore, we tried another simple baseline method that is described in the next section. The results concerning these methods were not shown above due to subpar performance compared to EB and XPN. Here we present the baseline normalization and the Performance Evaluation results for it and for all the three leading method including DWD and the comparison between them.

#### A.1 A baseline normalization method

In addition to the normalization methods mentioned earlier, we used brute force normalization as a baseline for comparison. We define a dataset *X* as the reference dataset, and all other datasets are normalized towards it. The choice of *X* can be arbitrary, but ideally, it should be the largest and most representative dataset. We calculate the mean sample vector of dataset *X* and sort it to create a new scale of measurements. Then, for each dataset, for each sample, we assign each entry a new value according to its rank in the reference scale of measurements, and by that all expression values are rescaled so that any RNA-seq sample contains exactly the same set of values. Here we used dataset GSE60424 as the reference dataset for all normalization of human datasets and cross-species normalization in which this dataset was part of, GSE107011 for other cross-species normalizations in which GSE60424 was not part of, and GSE122597 for normalization of mouse datasets. Our baseline method is quite similar to the quantile normalization method in enforcing the same set of values for all samples. The difference between the two methods is that, in the baseline method used here, this set of values is defined by the mean sample of the reference dataset whereas in quantile normalization this set of values is defined by the mean of the sorted samples, i.e. the mean of the largest values, the mean of the second largest values etc.

#### A.2 Baseline and DWD results

**Fig 10:**
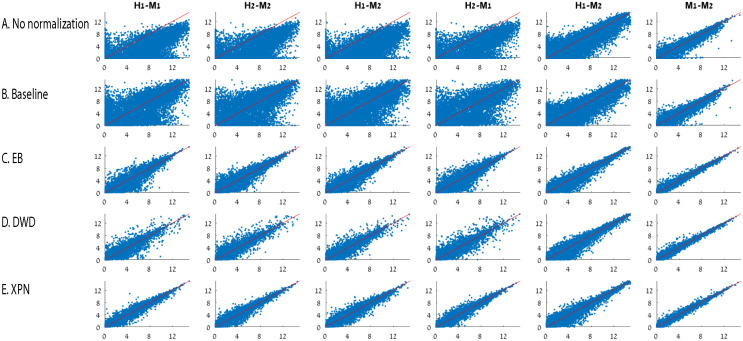
Comparison of overall similarity of samples. We compare log2 expression values before and after applying normalization methods using mean-mean scatter plots. (A) No normalization, (B) Baseline normalization, (C) EB [9], (D) DWD [7] and (E) XPN [6]. Mean-mean scatter plots of naïve CD4 T cells from different datasets: H1 is the human dataset GSE60424, M1 is the mouse dataset GSE122597, H2 is the human dataset GSE107011 and M2 is mouse dataset GSE12482. In each scatter plot the mean vector of the first dataset is on the X axis and the mean vector of the second dataset is on the Y axis. The red line is the *y* = *x* equation. Axes represent log2 expression values.

In Fig 10, the reduction in technical noise can be reflected in the a decrease of distribution width of the genes around the main diagonal. By looking in Fig 10B, it is evident that the baseline normalization may increase the alignment of the expression values to the diagonal, but it did not reduce the technical noise expressed by the wide distribution of the scatter compared to other methods.

Fig 10D shows that although DWD did reduce the distribution width of the genes around the diagonal, the application of the method on human-mouse studies resulted in a wider distribution of values throughout the diagonal compared to EB and XPN.

**Fig 11:**
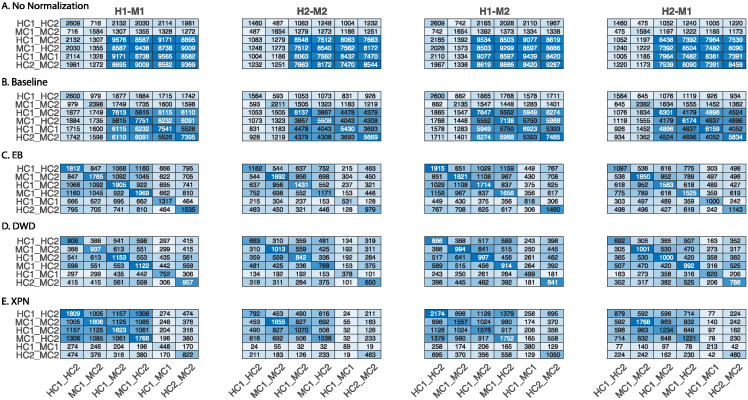
Comparison of lists of DEGs before and after normalizations. Comparison of lists of DEGs before and after applying four normalization methods on cross-species datasets. (A) No normalization, (B) Baseline, (C) EB, (D) DWD and (E) XPN. Shown is the intersection size between the species lists, the condition lists and the cross lists. The diagonal entries are the intersection size of each list with itself, i.e. every diagonal entry (i,i) is the number of genes in list i. Entry (i,j) represents the number of genes in the intersection of list i with list j. H1 is the human dataset GSE60424, M1 is the mouse dataset GSE122597, H2 is the human dataset GSE107011 and M2 is mouse dataset GSE124829. The condition C1 is CD4 T cells and the condition C2 is B cells.

This trend persists in Fig 11: both baseline method and DWD performed poorly. Similar to non-normalized datasets, the baseline method produced higher values in species lists, cross lists and their intersections than any other entry. This is evident by a dark homogeneous square on the lower right corner in Fig 11B. These large intersection likely taken place due to the technical difference between the datasets.

**Fig 12:**
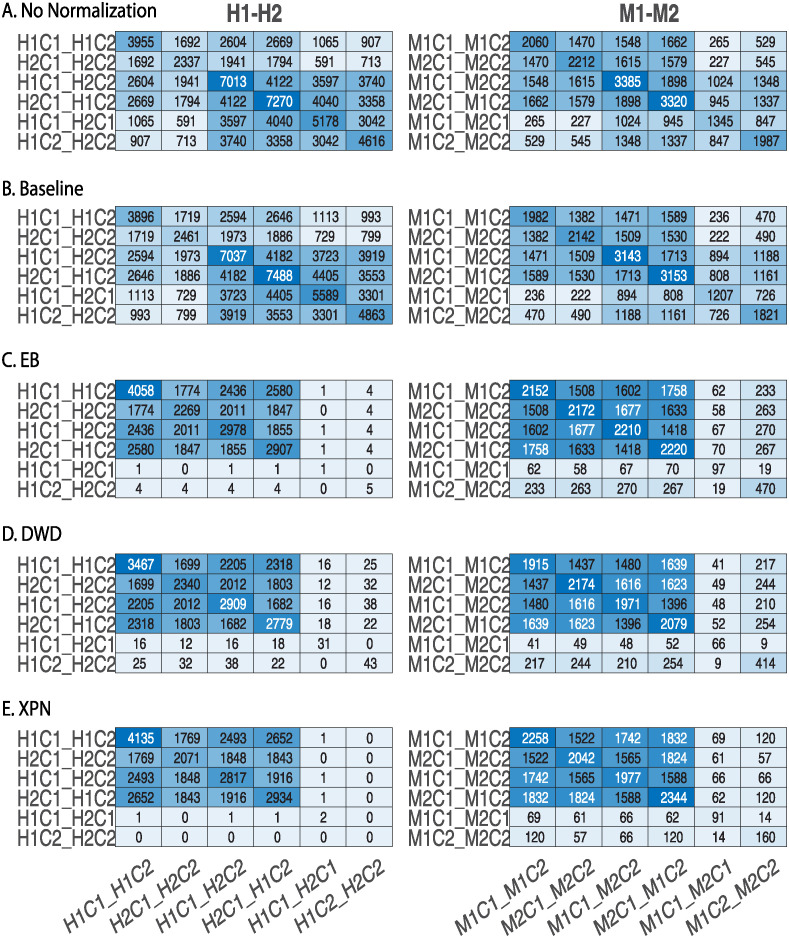
Comparison of lists of differentially expressed genes before and after applying four normalization methods on same species datasets. (A) No normalization, (B) Baseline, (C) EB, (D) DWD and (E) XPN. Shown is the intersection size between the species lists, the condition lists and the cross lists. The diagonal entries are the intersection size of each list with itself, i.e. every diagonal entry (i,i) is the number of genes in list i. Entry (i,j) represents the number of genes in the intersection of list i with list j. H1 is the human dataset GSE60424, M1 is the mouse dataset GSE122597, H2 is the human dataset GSE107011 and M2 is mouse dataset GSE124829. The condition C1 is CD4 T cells and the condition C2 is B cells.

DWD preformed better than the baseline method but worse than EB and XPN: as shown in Fig 11D. the number of genes in the condition lists decreased greatly compared to EB and XPN, indicating inner study information regarding the DEGs between conditions was lost, making this method less reliable for DEG analysis. As expected, the application of the normalization methods on the same species datasets resulted in better performance (Fig 12).

**Fig 13:**
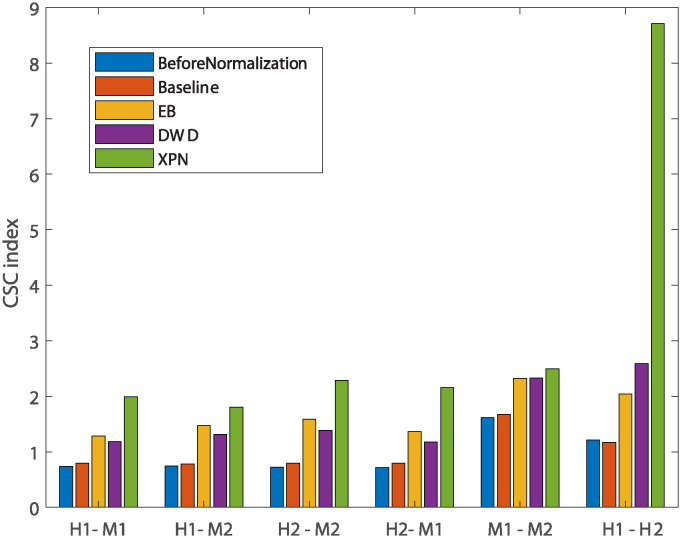
Comparisons of performance of normalization methods according to the CSC index. Every bar represents the CSC index for a specific comparison under a specific normalization method applied on the two datasets. H1 is the human dataset GSE60424, M1 is the mouse dataset GSE122597, H2 is the human dataset GSE107011 and M2 is the mouse dataset GSE124829.

**Fig 14:**
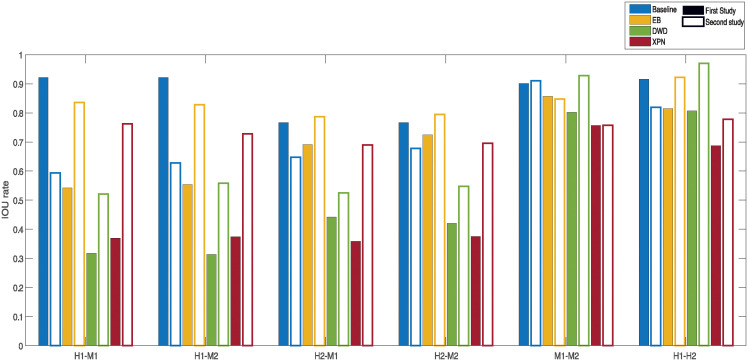
Comparisons of performance of normalization methods according to the IOU index. Every bar represents the IOU index for a condition list after normalization. A full bar represents the condition list from the first study and an empty bar represents the condition list from the second study. H1 is the human dataset GSE60424, M1 is the mouse dataset GSE122597, H2 is the human dataset GSE107011 and M2 is the mouse dataset GSE124829.

Fig 13 shows the CSC index rate: firstly, a small increase in similarity between baseline and non-normalized datasets, and secondly, poor performance of the DWD method compared to EB and XPN. For same species dataset DWD had a higher CSC index than EB and lower than XPN, indicating that this method may be better suited for same species normalization than for cross species. This hypothesis reinforced by Table 2, where DWD has the lowest average score for cross species and the highest for same species normalization among the three leading methods. As a matter of fact, this trend can also be seen by Fig 14—in every pair of cross species datasets, both of the datasets had the lowest IOU index scores while for same species it performed well.

**Table 2:** Average IOU values across all condition lists created for each normalization method.

Type of average IOU value	Baseline	EB	DWD	XPN
All condition lists	0.79	0.77	0.6	0.61
Cross-species condition lists	0.74	0.72	0.46	0.54
Condition lists within species	0.89	0.86	0.88	0.75

Not surprisingly, the baseline normalization has the highest average IOU values as shown in Table 2. The reason for such a phenomenon is that the reference dataset stays similar after the normalization. This is a clear example of how the IOU average score is not enough—while the reference datasets have a score close to 1, the other datasets score very low as can be seen in Fig 14.

### B Optimization using CMA-ES

**Fig 15:**
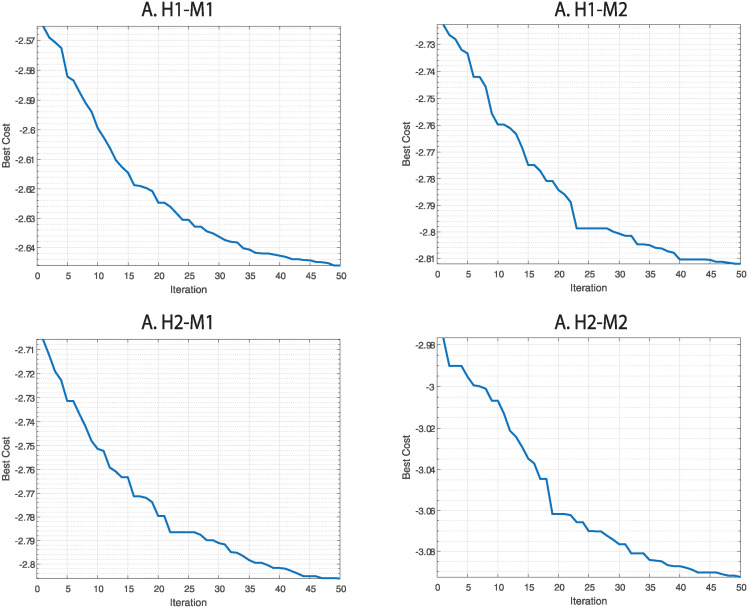
CMA-ES performance during the iterations of the normalization method according to the objective function with *t* = 0. Every graph represents the performance of the CMA-ES algorithm during its iterations. H1 is the human dataset GSE60424, M1 is the mouse dataset GSE122597, H2 is the human dataset GSE107011 and M2 is the mouse dataset GSE124829.

In Fig 15 we can see the objective function value during the iterations of the CMA-ES algorithm. It is clear that this algorithm reduces the cost given by the objective function. This phenomenon can be seen in any normalization applied on every two datasets. By that we can understand that the gradient free optimization algorithm we chose was effective for our goal.
